# GPATCH3 negatively regulates RLR-mediated innate antiviral responses by disrupting the assembly of VISA signalosome

**DOI:** 10.1371/journal.ppat.1006328

**Published:** 2017-04-17

**Authors:** Ying Nie, Yong Ran, Hong-Yan Zhang, Zhe-Fu Huang, Zhao-Yi Pan, Su-Yun Wang, Yan-Yi Wang

**Affiliations:** 1 Wuhan Institute of Virology, Key Laboratory of Special Pathogens and Biosafety, Chinese Academy of Sciences, Wuhan, Hubei, China; 2 University of Chinese Academy of Sciences, Beijing, China; University of Southern California, UNITED STATES

## Abstract

Upon viral infection, retinoic acid–inducible gene I–like receptors (RLRs) recognize viral RNA and trigger a series of signaling events, leading to the induction of type I interferons (IFNs). These processes are delicately regulated to prevent excessive and harmful immune responses. In this study, we identified G patch domain-containing protein 3 (GPATCH3) as a negative regulator of RLR-mediated antiviral signaling pathways. Overexpression of GPATCH3 impaired RNA virus- triggered induction of downstream antiviral genes, whereas its knockdown had opposite effects and attenuated viral replication. In addition, GPATCH3-deficient cells had higher *IFNB1* mRNA level compared with control cells after RNA virus infection. Mechanistically, GPATCH3 was recruited to VISA in a viral infection dependent manner and the assembly of VISA/TRAF6/TBK1 signalosome was impaired in GPATCH3-overexpressing cells. In contrast, upon viral infection, the recruitment of TRAF6 and TBK1 to VISA was enhanced in GPATCH3 deficient cells. Taking together, our findings demonstrate that GPATCH3 interacts with VISA and disrupts the assembly of virus-induced VISA signalosome therefore acts as a negative regulator of RLR-mediated innate antiviral immune responses.

## Author summary

Virus infection triggers the host cells to produce type I IFNs and proinflammatory cytokines, which are secreted proteins important for the host to clear viruses. Previously, we identified VISA (also named as MAVS, IPS-1 and Cardif) as a critical adaptor of virus-triggered, RLR-mediated induction of innate antiviral responses. In this study, we further found that GPATCH3, a functionally uncharacterized protein, interacted with mitochondria-localized VISA upon virus infection and disrupted the assembly of VISA-signalosome. Therefore, GPATCH3 acts as a negative regulator of VISA and functions as a brake of RLR-mediated antiviral innate responses. This discovery helps to understand how the innate antiviral responses are delicately regulated.

## Introduction

Recognition of conserved molecular structures of viruses by the host pattern-recognition receptors (PRRs) initiates innate antiviral immune responses. Several families of PRRs, including Toll-like receptors (TLRs), RIG-like receptors (RLRs), NOD-like receptors (NLRs) and recently identified DNA sensors, have been shown to sense different microbial components [1–3]. It has been demonstrated that cytosolic RNAs derived from viral genome or its replication intermediates are mainly recognized by the RLR family members retinoic acid–inducible gene-1 (RIG-I) and melanoma differentiation-associated gene 5 (MDA5) [4, 5]. The binding of RLRs to viral RNAs leads to rapid activation of transcription factors IRF3 and NF-κB, which collaborate to induce transcription of type I interferon (IFN) genes. Type I IFNs then activates JAK-STAT signaling pathways to induce the transcription of a wide range of interferon stimulated genes (ISGs) leading to innate antiviral responses [6].

Upon recognition of viral RNA, RIG-I and MDA5 undergo conformational changes and are recruited to the downstream mitochondria-associated adaptor protein VISA (also known as MAVS, IPS-1, Cardif) [7–11]. VISA then forms large prion-like polymers and serves as platform to assemble VISA signalosome by recruiting multiple components, including TRAF proteins (TRAF2/3/5/6), TBK1 and IKK kinases [12–14]. VISA recruits the TRAF proteins through its TRAF-binding motifs [7, 9, 15, 16], which in turn recruit TBK1 and IKKs to the VISA-associated signalosome, in which TBK1 and IKKs phosphorylate IRF3 and NF-κB, respectively leading to the induction of type I IFNs and proinflammatory cytokines [12, 13]. In addition to mitochondria, VISA has also been found to localize to peroxisomes [17]. Upon viral infection, peroxisomal VISA rapidly mounts a short-term protection by initiating the production of type III IFNs [18].

Several studies have demonstrated that dysregulation of RLR signaling is associated with autoimmune diseases [19–22]. For example, loss of function of MDA5 is associated with resistance to type I diabetes, whereas gain of function of MDA5 leads to spontaneously developed lupus-like nephritis and systemic autoimmune symptoms in mouse model [21, 23]. As a central adaptor of RLR-mediated signaling, VISA is expected to be tightly regulated by host factors [24]. It has been shown that WDR5 plays an important role in the assembly and stability of VISA signalosome [25]whereas TOM70 and IFIT3 facilitate VISA-mediated signaling by linking TBK1 to VISA [26, 27]. In contrast, it has been demonstrated that the virus-induced protein UBXN1 inhibits VISA-mediated signaling by interfering the assembly VISA signalosome [28]. In this study, we identified G patch domain-containing protein 3 (GPATCH3) as a negative regulator of RLR-mediated innate antiviral responses. We found that deficiency of GPATCH3 significantly enhanced RNA virus-triggered induction of type I IFNs and attenuated viral replication. Biochemically, GPATCH3 was recruited to VISA in a viral infection dependent manner where it disrupts the assembly of VISA/TRAF6/TBK1 complexes. Our findings suggest that GPATCH3 negatively regulates the assembly of VISA signalosome and shed first light on the biological function of GPATCH3.

## Results

### Identification of GPATCH3 as a negative regulator of RLR-mediated signaling

**Fig 1:**
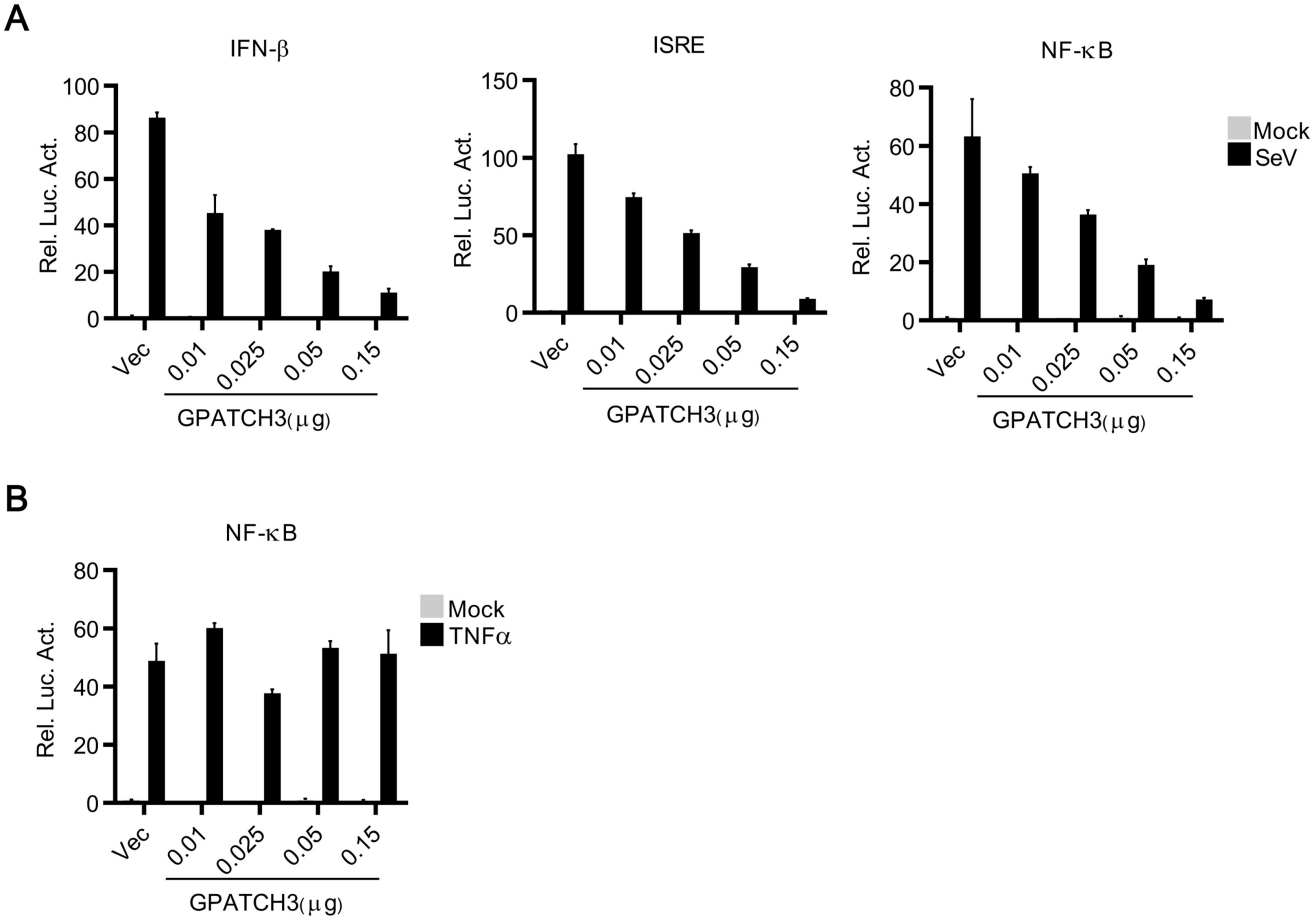
Overexpression of GPATCH3 inhibits RLR-mediated signaling. (A) 293T cells (1 x 10^5^) were cotransfected with empty vectors or increased amounts of GPATCH3 expression plasmids together with indicated luciferase reporters (0.05 μg each). Twenty-four hours after transfection, cells were stimulated with SeV for 12 hours before luciferase assays were performed. (B) The experiments were similarly performed as in A, except that cells were stimulated with TNF α (20 ng/ml). Graphs show mean ± SD. n = 3. *P<0.05, **P<0.01 (Student’s *t*-test).

RLRs recognize viral RNAs and initiate signal transductions leading to the induction of innate antiviral immune responses. To identify potential molecules involved in RLR-mediated signaling, we screened ~10,000 human cDNA expression plasmids for their abilities to regulate SeV-triggered induction of type I IFNs by luciferase assays. These efforts led to the identification of GPATCH3, a G-patch domain containing protein, as a negative regulator of RLR-mediated signaling (S1A Fig). GPATCH3 is ubiquitously expressed in mammalian tissues (EST profile from NCBI). However, the function of GPATCH3 has not yet been characterized. In reporter assays, overexpression of GPATCH3 reduced SeV-triggered activation of IFN-β promoter, ISRE and NF-κB in a dose-dependent manner in 293T cells (Fig 1A). However, in similar experiments, overexpression of GPATCH3 showed little effects on TNFα-triggered activation of NF-κB even at high dosage (Fig 1B). These data indicate that GPATCH3 specifically inhibits RLR-mediated signaling.

### Deficiency of GPATCH3 potentiates RLR-mediated signaling

**Fig 2:**
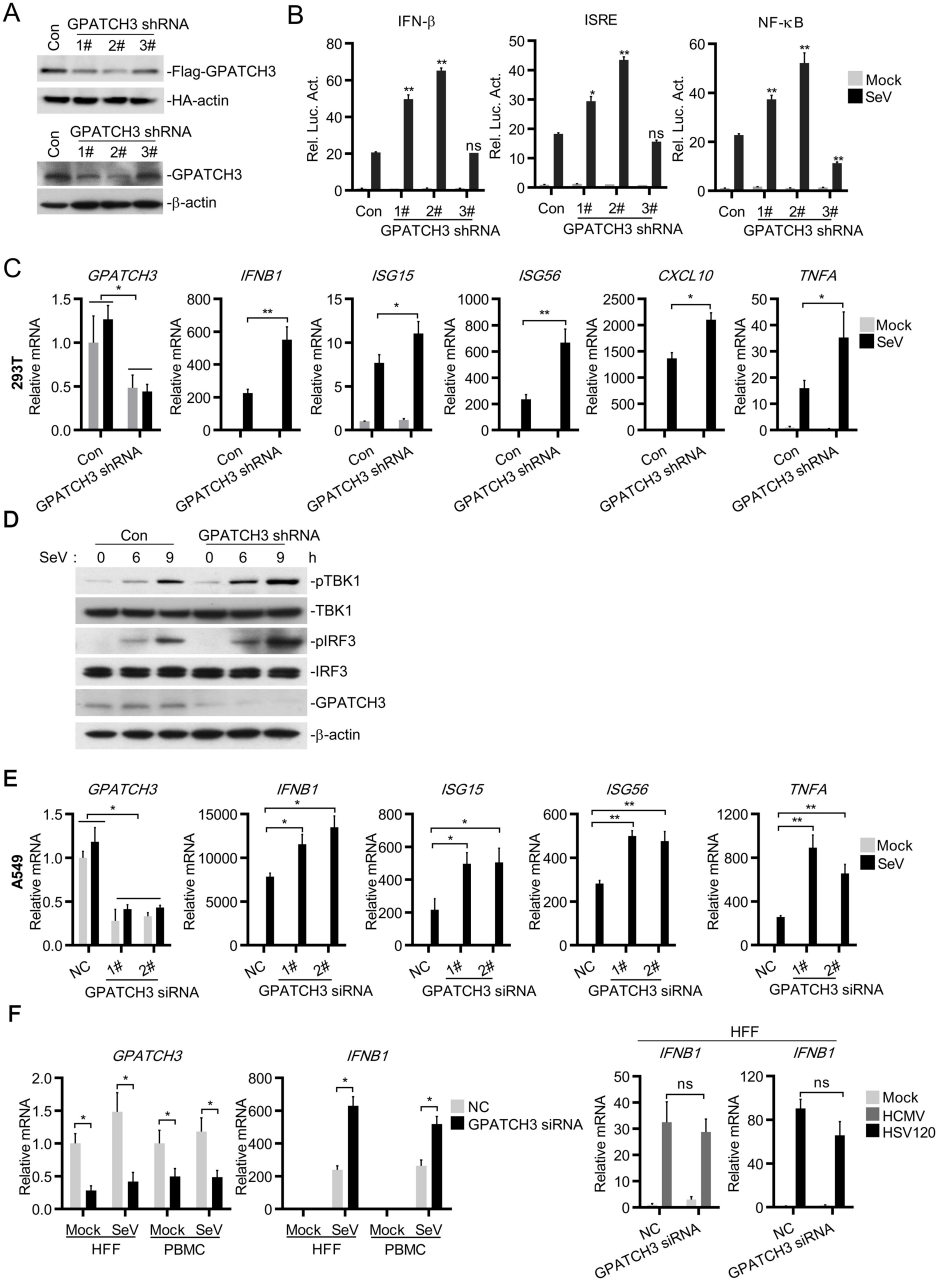
Knockdown of GPATCH3 enhances the activation of RLR-mediated signaling. (A) 293T cells (4 x 10^5^) were cotransfected with Flag-GPATCH3, HA-actin (0.1 μg each) and control- or the indicated GPATCH3-shRNA plasmids (1 μg each). Twenty-four hours after transfection, cell lysates were analyzed by immunoblotting with anti-Flag or anti-HA antibodies (upper panel). 293T cells (4 x 10^5^) were transfected with control- or the indicated GPATCH3-shRNA plasmids (1 μg each). Thirty-six hours after transfection, cell lysates were analyzed by immunoblotting with anti-GPATCH3 or anti-β-actin antibodies (lower panel). (B) 293T cells (1 x 10^5^) were transfected with control- or the indicated GPATCH3-shRNA plasmids (0.25 μg each) together with the indicated reporters (0.05 μg each). Thirty-six hours later, cells were left uninfected or infected with SeV for 12 hours before luciferase assays were performed. (C) 293T cells (4 x 10^5^) were transfected with control- or GPATCH3-shRNA plasmids (1 μg each). Thirty-six hours later, cells were left uninfected or infected with SeV for 12 hours before total RNAs were extracted and the mRNA levels of the indicated genes were analyzed by qPCR. (D) 293T cells (4 x 10^5^) were transfected with control- or GPATCH3-shRNA plasmids (1 μg each). Thirty-six hours later, cells were left uninfected or infected with SeV for the indicated times. Cell lysates were then analyzed by immunoblotting with the indicated antibodies. (E&F) A549 cells (4 x 10^5^), HFFs (4 x 10^5^) and PBMCs (1 x 10^6^) were transfected with control or GPATCH3 siRNAs (40 nM). Thirty-six hours later, cells were left untreated or treated with the indicated infections or transfections. Twelve hours later, total RNAs were extracted and the mRNA levels of the indicated genes were analyzed by qPCR. Graphs show mean ± SD. n = 3. *P<0.05, **P<0.01 (Student’s *t*-test).

To further investigate the roles of endogenous GPATCH3 in RLR-mediated signaling, we constructed three GPATCH3-shRNA plasmids targeting different sequences of human *GPATCH3* mRNA and examined their effects on the expression of GPATCH3. As shown in Fig 2A, three GPATCH3-shRNA constructs could knockdown the expression of GPATCH3 to different levels. The 2# GPATCH3-shRNA markedly inhibited the expression of GPATCH3, whereas the 1# and 3# GPATCH3-shRNA had partial or little effects on the expression of GPATCH3 respectively. In reporter assays, knockdown of GPATCH3 significantly potentiated SeV-induced activation of the IFN-β promoter, ISRE and NF-κB. Notably, the degrees of positive regulation of the IFN-β promoter, ISRE and NF-κB activation were correlated with the knockdown efficiencies of the respective shRNA plasmids (Fig 2B). We then used the 2# GPATCH3-shRNA for the following experiments. Similar results were obtained with the 1# GPATCH3-shRNA. Consistent with the results of reporter assays, quantitative PCR (qPCR) experiments showed that knockdown of GPATCH3 significantly enhanced SeV-induced transcription of *IFNB1*, *ISG15*, *ISG56*, *CXCL10* and *TNFA* (Fig 2C). Furthermore, knockdown of GPATCH3 enhanced SeV-induced phosphorylation of TBK1 and IRF3 (Fig 2D), which are hallmarks of activation of the RLR-mediated signaling. These data suggest that endogenous GPATCH3 negatively regulates RLR-mediated signaling.

To avoid the potential off-target effects of the GPATCH3-shRNA, we then performed reconstitution experiments. We generated a shRNA-resistant mutant of GPATCH3 (mGPATCH3) in which silent mutations were introduced into the targeting sequence of the GPATCH3-shRNA without changing the amino acid sequence. In reporter assays, the potentiation of SeV-induced activation of the IFN-β promoter, ISRE and NF-κB by the GPATCH3-shRNA was restored by the mGPATCH3 mutant (S2A Fig), indicating that the enhancement of RLR-mediated signaling was indeed caused by knockdown of GPATCH3.

To further confirm the function of endogenous GPATCH3 in other cell types, we generated two GPATCH3 siRNAs with different targeting sequences from GPATCH3-shRNAs and examined whether knockdown of GPATCH3 affects SeV-triggered induction of type I IFNs in A549 cells as well as in primary cells such as human foreskin fibroblasts (HFFs) and peripheral blood mononuclear cells (PBMCs). Consistently, knockdown of GPATCH3 significantly enhanced SeV-induced transcription of *IFNB1*, *ISG15*, *ISG56* and *TNFA* in A549 cells (Fig 2E). Moreover, knockdown of GPATCH3 potentiated SeV-induced transcription of *IFNB1* in HFFs and PBMCs (Fig 2F, left two panels). In contrast, knockdown of GPATCH3 had little effects on DNA virus HCMV- or double strand DNA HSV120-triggered transcription of *IFNB1* (Fig 2F, right panel). In addition, when cotransfected with cGAS and MITA (also known as STING), GPATCH3 had no significant effects on DNA sensor cGAS-mediated activation of ISRE and NF-κB (S2B Fig).

Since SeV-triggered induction of type I IFNs can be mediated by both RIG-I and MDA5, we next investigated the roles of GPATCH3 in RIG-I- and MDA5-mediated signaling. In reporter assays, knockdown of GPATCH3 markedly potentiated activation of the IFN-β promoter, ISRE and NF-κB triggered by transfected low and high molecular weight poly(I:C) (S2C & S2D Fig). It has been reported that RIG-I and MDA5 selectively recognize low and high molecular weight poly(I:C) respectively [29]. Therefore, these data suggest that GPATCH3 negatively regulates both RIG-I- and MDA5-mediated signaling. As a control, knockdown of GPATCH3 had no effects on TLR3-mediated signaling which was induced by poly(I:C) added to the cell culture media (S2E Fig). Taken together, these data reveal critical negative regulatory roles of GPATCH3 in RLR- but not DNA sensor- or TLR3-mediated signaling.

**Fig 3:**
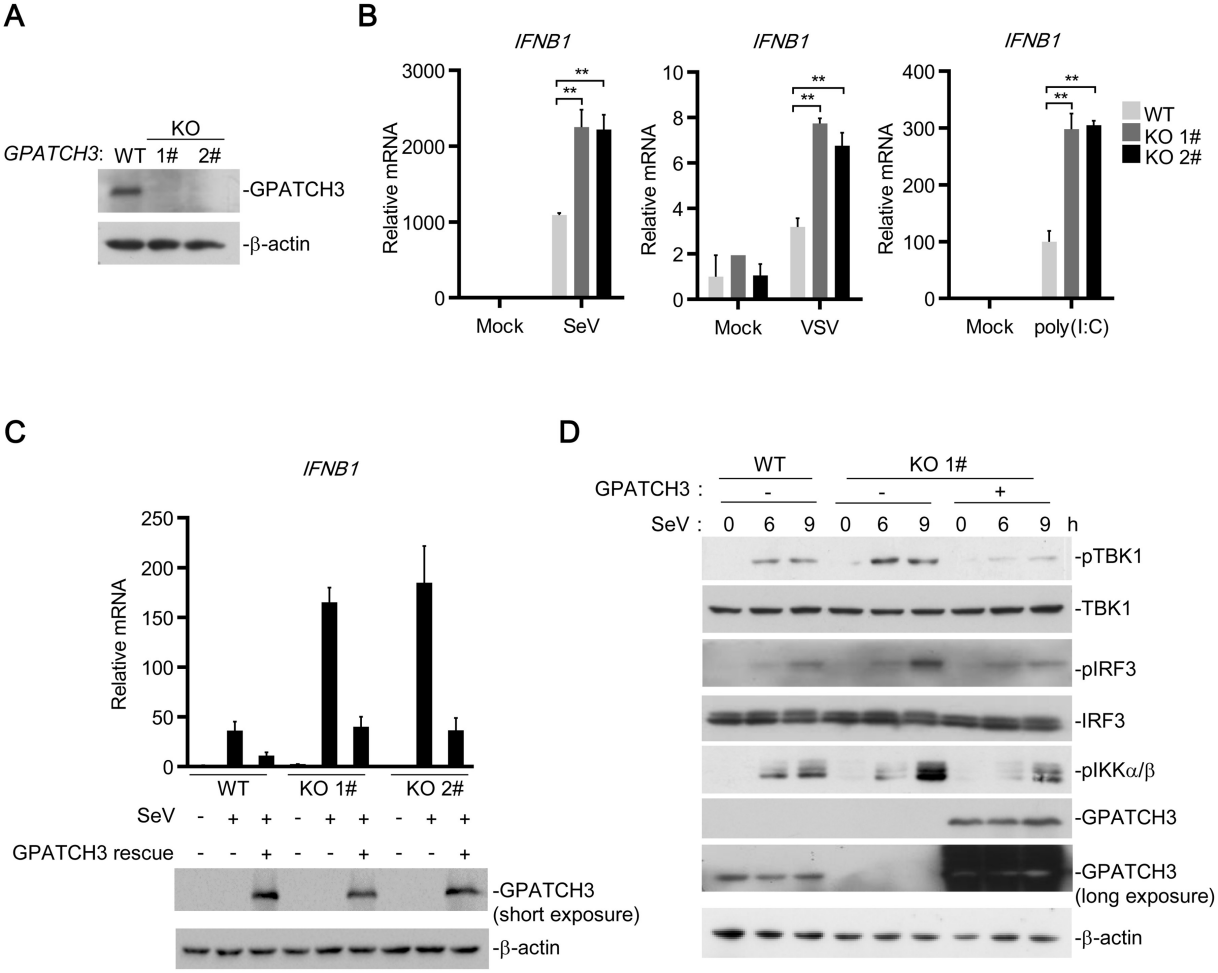
GPATCH3 deficiency potentiated RLR-mediated signaling. (A) *GPATCH3*-/- 293T cells were generated by CRISPR-Cas9 technology. Two GPATCH3 knockout clones and one wild-type clone (1 x 10^6^) were analyzed by immunoblotting with the anti-GAPTCH3 antibody. (B) Wild-type or *GPATCH3*-/- cells (1 x 10^6^) were left uninfected, infected with SeV, VSV (MOI = 0.1) or transfected with poly(I:C) (1 μg/ml). Twelve hours later, total RNAs were extracted and the mRNA level of *IFNB* was analyzed by qPCR. (C) Wild-type (WT) or GPATCH3-deficient cells (KO 1#&2#) (4 x 10^5^) were transfected with control or GPATCH3 expression plasmids (1 μg). Twenty-four hours after transfection, cells were left uninfected or infected with SeV. Twelve hours later, total RNAs were extracted and the mRNA level of *IFNB* was analyzed by qPCR. Expression of transfected GPATCH3 was detected by immunoblotting with anti-GPATCH3 antibody. (D) Wild-type (WT) or GPATCH3-deficient cells (KO 1#) (4 x 10^5^) were transfected with empty vector or GPATCH3 expression plasmids (1 μg). Cells were left uninfected or infected with SeV for the indicated times. Immunoblotting was then performed with the indicated antibodies. Graphs show mean ± SD. n = 3. *P<0.05, **P<0.01 (Student’s *t*-test).

To further confirm these results, we generated GPATCH3-deficient 293T cells by CRISPR-Cas9 technology using a gRNA targeting the exon 1 of the *Gpatch3* gene. Knockout of GPATCH3 was confirmed in two independent 293T clones (GPATCH3-KO 1#&2#) by immunoblotting analysis (Fig 3A). As shown in Fig 3B, compared with the control cells, GPATCH3-deficiency significantly enhanced transcription of *IFNB1* induced by SeV, VSV and cytosolic poly(I:C). Intriguingly, when we reconstituted the expression of GPATCH3 in GPATCH3-deficient cells, the over-activated transcription of *IFNB1* was restored (Fig 3C). Compared with the knockout cells, reconstitution of GPATCH3 into wild type cells resulted in lower induction of *IFNB1* because the total amount of GPATCH3 in wild type cells, which endogenously express GPATCH3, was higher than that of the knockout cells (Fig 3C). The 1# GPATCH3-KO clone was further used to detect SeV-induced phosphorylation of TBK1, IRF3 and IKK α/β, which are hallmarks of their activation. As shown in Fig 3D, knockout of GPATCH3 markedly increased SeV-induced phosphorylation of TBK1, IRF3 and IKKα/β whereas reconstitution of GPATCH3 in the knockout cells reversed such increases. These data indicate that GPATCH3 plays an important role in the negative regulation RLR-mediated signaling.

### GPATCH3 inhibits cellular antiviral responses

**Fig 4:**
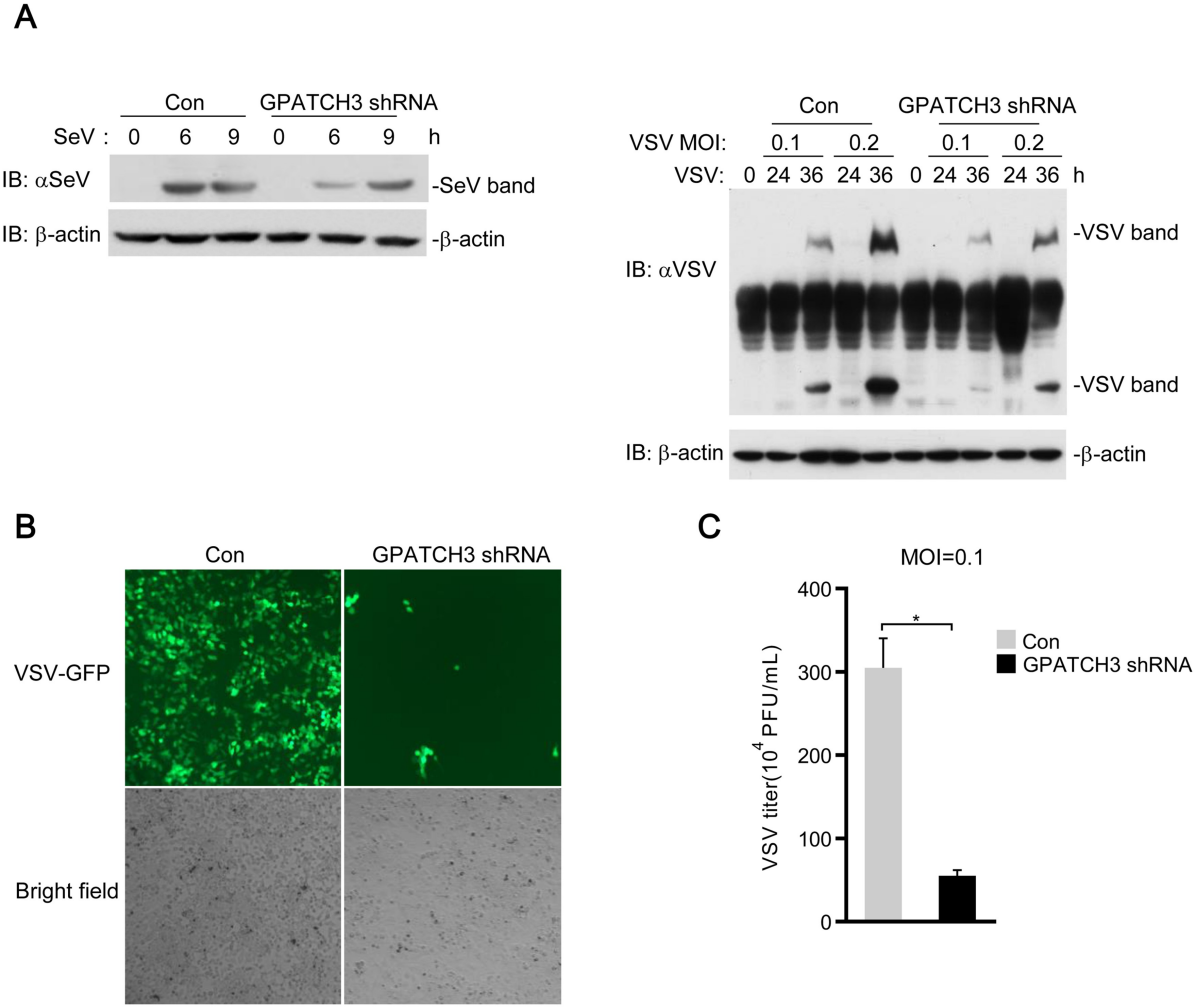
GPATCH3 negatively regulates the cellular antiviral responses. (A) 293T cells (4 x 10^5^) were transfected with control- or GPATCH3-shRNA plasmids (1 μg). Thirty-six hours later, cells were left uninfected or infected with SeV or indicated mounts of VSV for the indicated times. Cell lysates were analyzed by immunoblotting with antibodies against virus proteins. β-actin was immunoblotted as a control. (B) 293T cells (4 x 10^5^) were transfected with control- or GPATCH3-shRNA plasmids (1 μg). Thirty-six hours after transfection, cells were infected with VSV-GFP (MOI = 0.1) for thirty-six hours. Cells were imaged by fluorescence microscopy. (C) 293T cells (4 x 10^5^) were transfected with control- or GPATCH-shRNA plasmids (1 μg). Thirty-six hours later, cells were transfected with poly(I:C) (1 μg/ml). Twelve hours later, cells were infected with VSV (MOI = 0.1) and supernatants were harvested 24 hours post-infection. Supernatants were then analyzed for VSV production by standard plaque assays. Graphs show mean ± SD. n = 3. *P<0.05, **P<0.01 (Student’s *t*-test).

Since GPATCH3 negatively regulates RLR-mediated induction of type I IFNs, we next examined whether GPATCH3 affected cellular antiviral responses. We measured replications of SeV and VSV by immunoblotting analysis using antibodies against viral proteins. As shown in S3A Fig, overexpression of GPATCH3 resulted in higher levels of SeV and VSV proteins. Consistently, knockdown of GPATCH3 markedly impaired replications of both SeV and VSV at all examined time points post infection (Fig 4A). To confirm these results, replication of VSV was further measured by immunofluorescence microscopy of VSVs tagged with GFP and plague assays. Data from these experiments showed that knockdown of GPATCH3 resulted in decreased replication of VSV, as indicated by the less green fluorescence (Fig 4B) as well as the lower virus titers (Fig 4C) in the GPATCH3-shRNA transfected cells, suggesting a more robust antiviral response in GPATCH3-knockdown cells. These observations suggest that GPATCH3 negatively regulates cellular antiviral responses.

### GPATCH3 targets VISA for its inhibitory function

**Fig 5:**
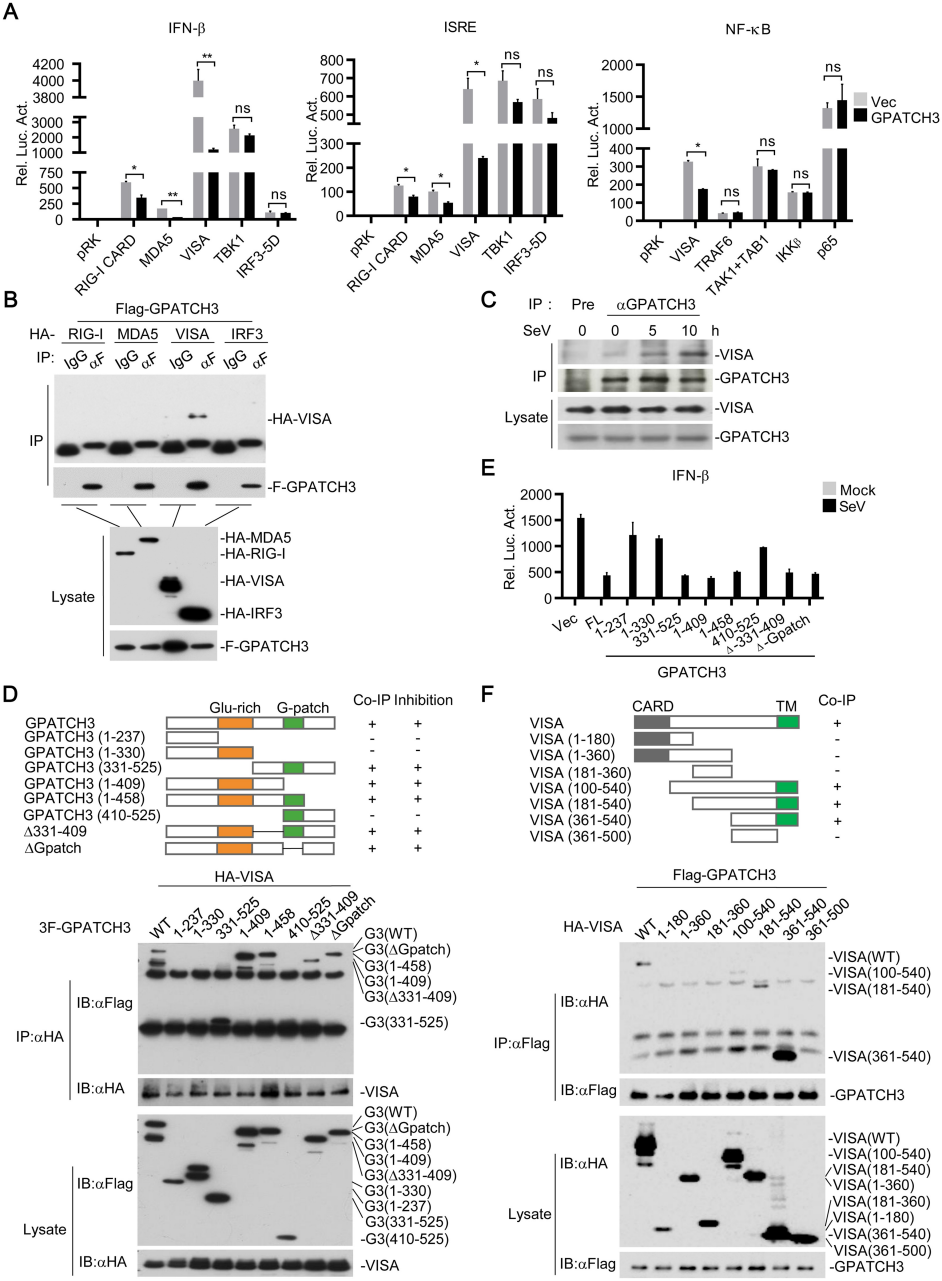
GPATCH3 interacts with VISA and inhibits VISA-mediated signaling. (A) 293T cells (1 x 10^5^) were cotransfected with the indicated reporters (0.05 μg each), expression plasmids (0.05 μg each) and empty vectors or GPATCH3 expression plasmids (0.05 μg). Luciferase assays were performed 24 hours after transfection. (B) 293T cells (2 x 10^6^) were cotransfected with Flag-GPATCH3 and HA-tagged RIG-I, MDA5, VISA or IRF3. Coimmunoprecipitation and immunoblotting were performed with the indicated antibodies. (C) 293T cells (1.5 x 10^7^) were left uninfected or infected with SeV for the indicated times. Coimmunoprecipitation and immunoblotting were performed with the indicated antibodies. (D) 293T cells (2 x 10^6^) were cotransfected with HA-VISA (3 μg) and the indicated Flag-tagged GPATCH3 truncations (3 μg). Coimmunoprecipitation and immunoblotting were performed with the indicated antibodies. (E) 293T cells (1 x 10^5^) were cotransfected with IFN-β reporter (0.05 μg) and the indicated GPATCH3 truncations (0.05 μg). Twenty-four hours later, cells were left uninfected or infected with SeV for 12 hours before luciferase assays were performed. (F) 293T cells (2 x 10^6^) were cotransfected with Flag-GAPTCH3 (3 μg) and the indicated HA-tagged VISA truncations (3 μg). Coimmunoprecipitation and immunoblotting were performed with the indicated antibodies. Graphs show mean ± SD. n = 3. *P<0.05, **P<0.01 (Student’s *t*-test).

To identify the potential regulatory targets of GPATCH3, we examined the effects of GPATCH3 on activation of the IFN-β promoter, ISRE and NF-κB mediated by components of the RLR signaling pathways. The results showed that cotransfection of GPATCH3 reduced activation of the IFN-β promoter, ISRE, and NF-κB mediated by VISA and its upstream components such as RIG-I-CARD and MDA5 but had no obvious effects on activation mediated by proteins downstream of VISA such as TBK1, IRF3-5D (a constitutively active mutant of IRF3), TRAF6, TAK1, TAB1, IKKβ or p65 (Fig 5A). Transient transfection and coimmunoprecipitation experiments showed that GPATCH3 interacted with VISA but not RIG-I, MDA5 or IRF3 (Fig 5B). Endogenous coimmunoprecipitation experiments indicated that GPATCH3 weakly interacted with VISA in un-infected cells, but their interaction was markedly increased following SeV infection (Fig 5C). These results suggest that GPATCH3 targets VISA for its inhibitory effects on RLR-mediated signaling.

We generated a series of GPATCH3 truncations and examined their abilities to interact with VISA. Domain mapping analysis indicated that no specific domain of GPATCH3 examined was responsible for its association with VISA (Fig 5D). A reasonable explanation is that a proper spatial conformation of full-length GPATCH3 is required for its binding to VISA. When tested for their abilities to inhibit VISA-mediated activation of the IFN-β promoter, only the truncations of GPATCH3 that interacted with VISA impaired VISA-mediated signaling (Fig 5E). Taken together, these date suggest that GPATCH3 interacts with VISA following viral infection and its physical binding to VISA is sufficient and necessary for the inhibitory function.

Domain mapping analysis was also performed to test which domains of VISA are responsible for its interaction with GPATCH3. As shown in Fig 5F, GPATCH3 associated with full-length VISA as well as truncations of VISA that contain the C-terminal transmembrane (TM) domain. In contrast, GPATCH3 failed to interact with VISA truncations lacking its TM domain. These results indicate that the TM of VISA is required for its binding with GPATCH3.

### GPATCH3 targets mitochondria-localized VISA

It has been reported that VISA is located on both mitochondrial and peroxisomal membranes through its C-terminal transmembrane domain [17, 18, 24]. Since the transmembrane domain of VISA is responsible for its interaction with GPATCH3, we next investigated whether GPATCH3 targets mitochondria or peroxisome localized VISA.

**Fig 6:**
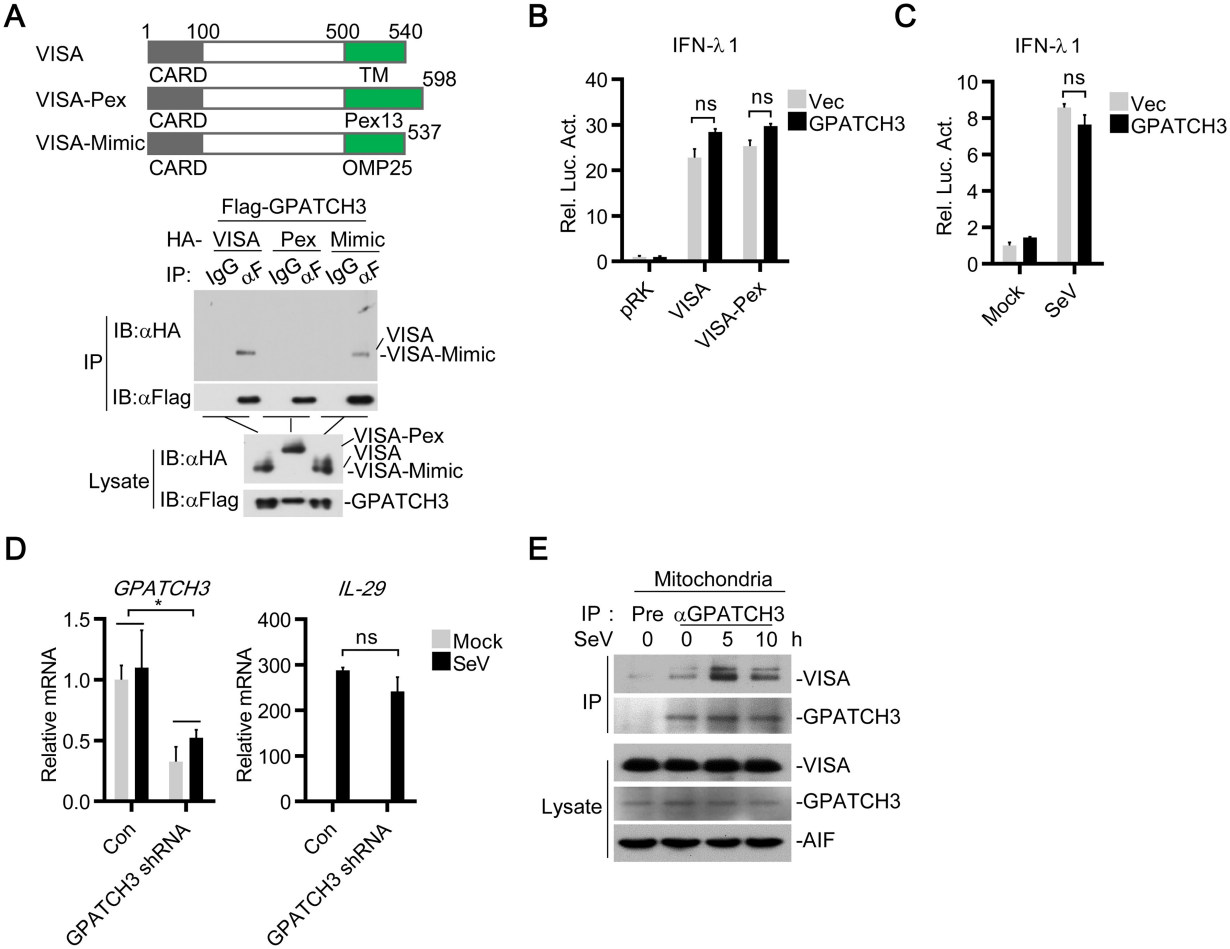
GPATCH3 targets mitochondria localized VISA. (A) 293T cells (2 x 10^6^) were cotransfected with Flag-GPATCH3 (3 μg) and HA-tagged VISA, VISA-Pex, or VISA-Mimic (3 μg). Coimmunoprecipitation and immunoblotting were performed with the indicated antibodies. (B) 293T cells (1 x 10^5^) were cotransfected with IFNλ1 reporter (0.05 μg), the indicated expression plasmids (0.05 μg each) and empty vectors or GPATCH3 expression plasmids (0.05 μg). Luciferase assays were performed 24 hours after transfection. (C) 293T cells (1 x 10^5^) were transfected with the IFNλ1 reporter (0.05 μg) together with empty vectors or GPATCH3 expression plasmids (0.05 μg). Twenty-four hours after transfection, cells were stimulated with SeV for 24 hours before luciferase assays were performed. (D) 293T cells (4 x 10^5^) were transfected with control- or GPATCH3-shRNA plasmids (1 μg). Thirty-six hours later, cells were left uninfected or infected with SeV for 6 hours before total RNAs were extracted and the mRNA levels of the indicated genes were analyzed by qPCR. (E) 293T cells (1.5 x 10^7^) were left uninfected or infected with SeV for the indicated times. Mitochondria were isolated by subcellular fractionation and the mitochondrial lysates were subjected to co-immunoprecipitation and immunoblotting analysis with the indicated antibodies. Graphs show mean ± SD. n = 3. *P<0.05, **P<0.01 (Student’s *t*-test).

We generated chimeric VISA expression plasmids by substituting the TM domain of VISA with the TM domain of Pex13 (VISA-Pex), a protein solely locates on peroxisomes, or with the TM domain of OMP25 (VISA-Mimic), a protein which was originally reported to localize on mitochondria [30]. Localizations of VISA-Pex to peroxisomes have already been demonstrated [17]. Unexpectedly, localization of VISA-Mimic have been found on both peroxisomes and mitochondria, which mimics the localization of wild-type VISA [17]. Transient transfection and coimmunoprecipitation experiments indicated that localization of VISA to peroxisomes completely abolished its binding to GPATCH3 (Fig 6A). However, VISA-Mimic could still interact with GPATCH3 (Fig 6A). These data suggest that GPATCH3 interacts with mitochondria-localized VISA.

Since mitochondrial and peroxisomal VISA have been suggested to mediate the production of type I and type III IFNs respectively[17], we attempted to determine whether GPATCH3 is involved in VISA-mediated production of type III IFNs. As shown in Fig 6B, overexpression of GPATCH3 had no significant effects on VISA or VISA-Pex- mediated activation of IFN-λ1 reporter. Moreover, overexpression of GPATCH3 did not affect SeV-induced activation of IFN-λ1 reporter (Fig 6C). Consistently, knockdown of GPATCH3 had no significant effects on SeV-induced transcription of *IL-29* (Fig 6D). Collectively, these data suggest that GPATCH3 is not involved in peroxisomal VISA-mediated induction of type III IFNs.

Since GPATCH3 specifically interacts with and inhibits the function of mitochondrial VISA, we next examined whether GPATCH3 localized on mitochondria by cellular fractionation assays. The results showed that GPATCH3 was localized in nucleus, cytosol and mitochondria (S4A Fig). Intriguingly, the subcellular distribution of GPATCH3 did not change after virus infection (S4A Fig). These data indicated that GPATCH3 was constitutively localized in mitochondria and was recruited to VISA after virus infection.

To further confirm these results, we isolated mitochondria by subcellular fractionation and performed endogenous coimmunoprecipitation experiments with mitochondrial lysates. Consistently, the results showed that although GPATCH3 constitutively localized on mitochondria, it was recruited to VISA in a virus infection dependent manner (Fig 6E). Collectively, these data suggest that GPATCH3 targets mitochondrial VISA to negatively regulate RLR-mediated production of type I IFNs.

### GPATCH3 disrupts the assembly of VISA signalosomes

We next investigated the mechanism of GPATCH3 mediated inhibition of VISA. Since aggregation of VISA is important for its activation, we first examined whether GPATCH3 inhibited VISA aggregation. As shown in S5A Fig, results of coimmunoprecipitation experiments showed that overexpression of GPATCH3 had no effects on VISA oligomerization which is the first step of VISA aggregation. Furthermore, we examined the role of endogenous GPATCH3 on VISA aggregation by Semi-Denaturating Detergent Agarose Gel Electrophoresis (SDD-AGE). Consistently, GPATCH3 deficiency had no significant effects on VISA aggregation (S5B Fig). Collectively, these data suggest that GPATCH3 does not target the activation of VISA.

**Fig 7:**
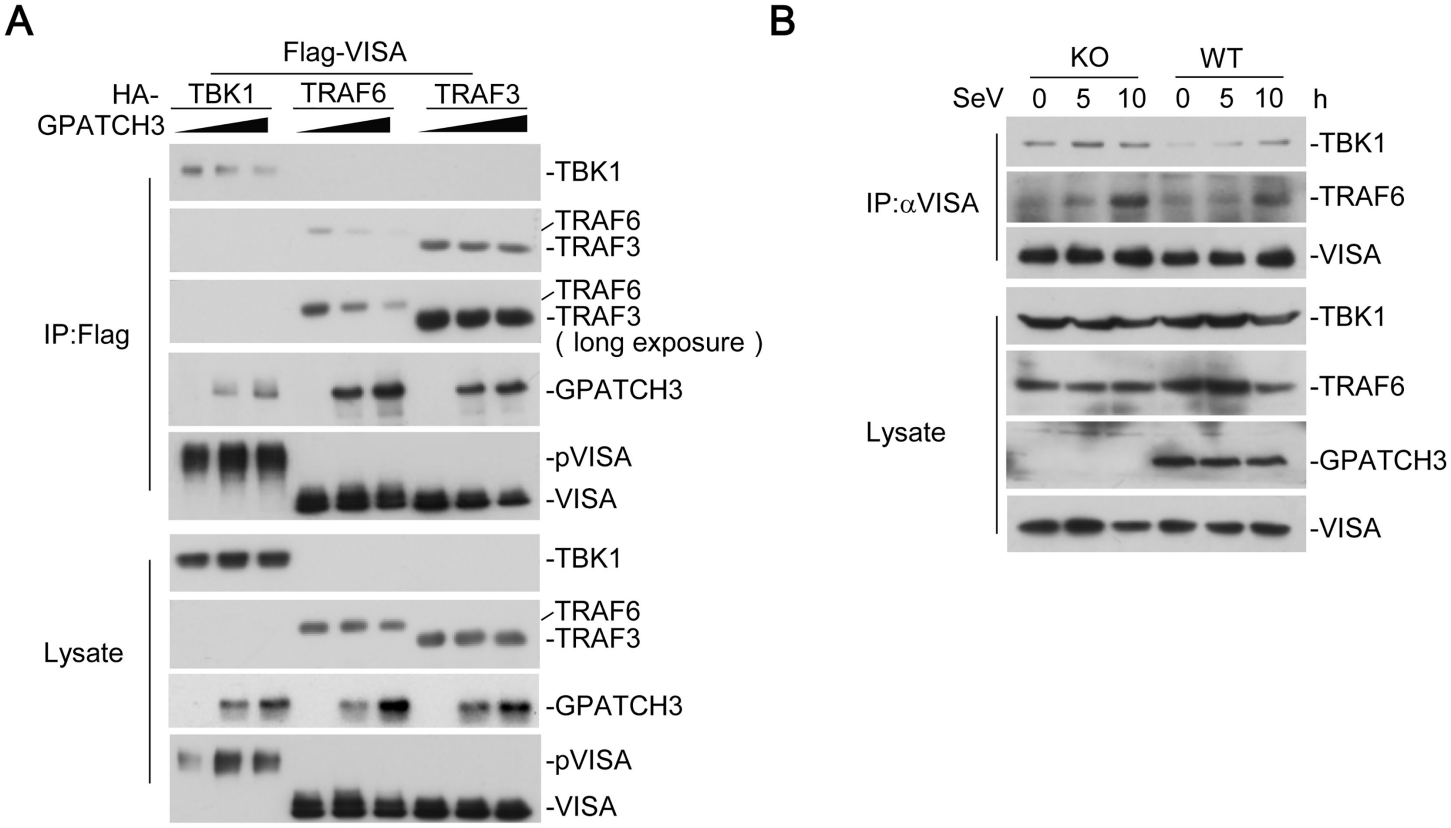
GPATCH3 interrupts assembly of the VISA-associated signaling complexes. (A) 293T cells (2 x 10^6^) were cotransfected with Flag-VISA (3 μg) and HA-tagged TBK1, TRAF6 or TRAF3 (3 μg), together with increasing amounts of GPATCH3 (0, 0.5 μg, 1.5μg). Coimmunoprecipitation and immunoblotting were performed with the indicated antibodies. (B) Wild-type or GPACH3-deficient cells (1.5 x 10^7^) were left uninfected or infected with SeV for the indicated times. Coimmunoprecipitation and immunoblotting were performed with the indicated antibodies.

We next determined whether GPATCH3 disrupted the association of VISA with downstream signaling components. We cotransfected VISA with its downstream signaling proteins such as TBK1, TRAF6 or TRAF3, together with an increased amount of GPATCH3. The results of coimmunoprecipitation experiments indicated that interactions of VISA-TBK1 and VISA-TRAF6 were markedly reduced by GPATCH3 in a dose dependent manner whereas the interaction of VISA-TRAF3 was not affected (Fig 7A). Furthermore, we examined the roles of endogenous GPATCH3 on assembly of the VISA signalosomes. Deficiency of GPATCH3 had no marked effects on the protein levels of VISA, TBK1 or TRAF6. However, compared with the wild-type cells, GPATCH3-deficiency markedly enhanced recruitment of TBK1 and TRAF6 to the VISA signalosomes (Fig 7B). These data suggest that GPATCH3 interferes with the assembly of VISA signalosomes, leading to the negative regulation of the RLR-mediated signaling.

## Discussion

The function of the G-patch domain containing protein GPATCH3 has been elusive. In this study, we identified GPATCH3 as a negative regulator of innate immune responses to RNA viruses. Knockdown of GPATCH3 significantly enhanced SeV-triggered induction of downstream antiviral genes in multiple cell lines, including primary PBMCs and HFFs but had no marked effects on TLR3- or DNA sensor-mediated signaling. GPATCH3-deficient cells showed higher induction of *IFNB1* compared with wild-type cells upon SeV or VSV infection. Mechanistically, GPATCH3 interacted with VISA and impaired assembly of the VISA-associated signaling complexes.

It has been demonstrated that VISA is essential for innate antiviral immune responses against to RNA viruses [31]. Previous studies suggest that VISA recruits TRAF3 and TRAF6 to activate IRF3 and NF-κB respectively [7, 9, 15]. However, recent gene knockout studies indicated that TRAF3 is dispensable for TBK1 and IRF3 activation whereas TRAF6 plays an important role to active both IRF3 and NF-κB [12, 32]. In this study, we found that GPATCH3 interrupted the binding of VISA to TRAF6 but not TRAF3 and inhibited RLR-mediated activation of both IRF3 and NF-κB, which is consistent with the conclusion of gene knockout studies. Interestingly, different from the previous discovery that UBXN1 negatively regulates the function of VISA by competing for the TRAF-binding motifs of VISA to block recruitment of TRAF3 and TRAF6 [28], our findings demonstrated that the binding of GPATCH3 to VISA and its blockade of assembly of the VISA-signalosome was independent of TRAF-binding motifs of VISA (aa143-147 for TRAF2/3/5, aa153-158 and aa455-460 for TRAF3/6) but dependent on the membrane localization of VISA.

VISA is located on mitochondria and peroxisomes and the distinct cellular localization leads to different types of antiviral responses [17, 18]. Mitochondria-localized VISA induces long term antiviral response through the expression of type I IFNs. In contrast, peroxisome-localized VISA induces the expression of type III IFNs. Notably, the C-terminal TM of VISA dictates its localization to different organelles [17, 24, 33]. In this study, we found that GPATCH3 specifically interacted with the mitochondrial VISA, which was sufficient and required for it to execute the inhibitory roles on RLRs-mediated signaling.

The G-patch domain-containing proteins are widely found in eukaryotes [34]. A previous study has shown that the G-patch containing protein MOS2 is essential for innate immunity in *Arabidopsis thaliana* [35]. Different from the function of MOS2 as a positive regulator of innate immunity, we found that GPATCH3 acted as a negative regulator of RLR-mediated innate antiviral responses. Notably, while we found that the interaction of GPATCH3 with VISA was essential for its inhibitory roles, the binding of GPATCH3 to VISA was independent of its G-patch domain and a GPATCH3 mutant lack of the G-patch domain inhibited SeV-induced activation of the IFN-β promoter to similar degrees as the full-length GPATCH3. These data suggested that the G-patch domain of GPATCH3 is not required for its inhibitory roles on innate antiviral responses. In conclusion, our findings suggest that GPATCH3-mediated disruption of VISA-associated complexes represents an important regulatory mechanism of innate antiviral responses.

## Materials and methods

### Cells, reagents, virus and antibodies

HFFs and HCMV were provided by Dr. Min-Hua Luo (Wuhan Institute of Virology, CAS). The 293 cells stably expressing TLR3 (293-TLR3) were provided by Katherine Fitzgerald (University of Massachusetts Medical school, Worcester, MA) and Tom Maniatis (Department of Molecular and Cellular Biology, Harvard University, Cambridge, MA). HEK293T (Transformed Human Embryonic Kidney 293 cell line, ATCC) cells, A549 (Human Lung Adenocarcinoma cell line, ATCC) cells, PBMCs (Peripheral Blood Mononuclear Cells, Allcells), Human recombinant TNFα (R&D Systems), poly(I:C) (InvivoGen), Lipofectamine 2000 (Invitrogen), dual-specific luciferase assay kits (Promega), mouse monoclonal antibodies against Flag and β-actin (Sigma), HA (Origene), AIF, LMNB1 and TBK1 (Abcam), IRF-3 (Proteintech), VISA (Bethyl Laboratories), TRAF6 and GPATCH3 (Y-20) (Santa Cruz Biotechnology), phospho-IRF3 (Ser396), phospho-TBK1 (Ser172) and phospho-IKKα (Ser176)/β (Ser177) (Cell Signaling Technology) were purchased from the indicated companies. Rabbit polyclonal anti-SeV and anti-VSV were previously described [36]. Mouse polyclonal antisera against GPATCH3 were raised against recombinant human C-terminal GPATCH3 fragment (aa 299–525).

### Constructs

IFN-β promoter, ISRE and NF-κB luciferase reporter plasmids, mammalian expression plasmids for HA- or Flag-tagged RIG-I-CARD, MDA5, VISA and its mutants, TBK1, IRF3, TRAF6, TRAF3, TAK1, TAB1, IKKβ, p65 were previously described [7, 36–38]. The IFN-λ1 luciferase reporter plasmid was provided by Dr. Ying Zhu (Wuhan University). Mammalian expression plasmids for human HA-, Flag-tagged GPATCH3 and its deletion mutants were constructed by standard molecular biology techniques.

### DNA oligonucleotides

HSV120: 5’-AGACGGTATATTTTTGCGTTATCACTGTCCCGGATTGGACACGGTCTTGTGGGATAGGCATGCCCAGAAGGCATATTGGGTTAACCCCTTTTTATTTGTGGCGGGTTTTTTGGAGGACTT-3’.

### Transfection and reporter gene assays

HEK293T cells or 293-TLR3 cells (1x10^5^) were seeded on 48-well plates and transfected the following day by standard calcium phosphate precipitation. Empty control plasmid was added to ensure that each transfection receives the same amount of total DNA. To normalize for transfection efficiency, 0.005 μg of pRL-TK Renilla luciferase reporter plasmid was added to each transfection. Luciferase assays were performed with a dual-specific luciferase assay kit (Promega). Firefly luciferase activities were normalized on the basis of Renilla luciferase activities.

### RNAi experiments

Double-strand oligonucleotides corresponding to the target sequences were cloned into the pSuper-Retro plasmid (Oligoengine). The following sequences were targeted for human GPATCH3 mRNA: 1#: 5’-GCAAGCGTGGATTGGGGTA-3’; 2#: 5’-CCTACCTGGCAGATATACC-3’; 3#: 5’-GTGAAGAAATACCCCAAGG-3’. Small interfering RNAs (siRNAs) targeting human GPATCH3 were purchased from Ribobio. 1# siRNA: 5’-GGAACAGAGACTCCGAGAT-3’, 2# siRNA: 5’-GTACCATGGAGAGAAGCTA-3’. siRNA were delivered into A549s and HFFs by PepMute siRNA transfection reagent (SignaGen) according to procedures recommended by the manufacturer.

### CRISPR-Cas9

Genome engineering using the CRISPR-Cas9 technology was previously described [39, 40]. pGL3-U6-gRNA and pST1374-Cas9-D10A plasmids were provided by Dr. Xiao-Dong Zhang (Wuhan University). GPATCH3 gRNA targeting sequence: 5’-CGCCGCGCTCTTCTCGGAAC-3’.

### Quantitative real-time PCR

Total RNA was isolated and reversed transcription to cDNA for quantitative real-time PCR analysis to measure the mRNA levels of tested genes. GAPDH was used as a reference gene. Human gene specific primer sequences were as follows: GAPDH: 5’-GAGTCAACGGATTTGGTCGT-3’ (forward), 5’-GACAAGCTTCCCGTTCTCAG-3’ (reverse); IFNB: 5’-TTGTTGAGAACCTCCTGGCT-3’ (forward), 5’-TGACTATGG TCCAGGCACAG-3’ (reverse); ISG15: 5’-AGGACAGGGTCCCCCTTGCC-3’ (forward), 5’- CCTCCAGCCCGCTCACTTGC-3’ (reverse); ISG56: 5’-TCATCAGGT CAAGGATAGTC-3’ (forward), 5’-CCACACTGTATTTGGTGTCTAGG-3’ (reverse); IP-10: 5’- GGTGAGAAGAGATGTCTGAATCC-3’ (forward), 5’- GTCCATCCTTGG AAGCACTGCA-3’ (reverse); TNFa: 5’-GCCGCATCGCCGTCTCCTAC-3’ (forward), 5’- CCTCAGCCCCCTCTGGGGTC-3’ (reverse); IL-29: 5’-CGCCTTGGAAGAGTCACTCA-3’ (forward), 5’-GAAGCCTCAGGTCCCAATTC-3’ (reverse); and GPATCH3: 5’-TGGCTGGATT CTCACGGGACTT-3’ (forward), 5’- GGTGAAGGCTTCATTCTCTGCC-3’ (reverse).

### Co-immunoprecipitation

Cells (5 x 10^6^ for overexpression experiments and 2 x 10^7^ for endogenous experiments) were lysed in l ml NP-40 lysis buffer (20 mM Tris-HCl [pH 7.4], 150 mM NaCl, 1mM EDTA, 1% Nonidet P-40, 10 mg/ml aprotinin, 10 mg/ml leupeptin, and 1 mM phenylmethylsulfonyl fluoride). Coimmunoprecipitation and immunoblot analysis were performed as previously described [41].

### Virus manipulation

These experiments were performed as described [42].

### Subcellular fractionation

The experiments were performed with subcellular fractionation kits (ApplyGene) following protocols recommended by the manufacture.

### Semi-Denaturing Detergent Agarose Gel Electrophoresis (SDD-AGE)

SDD-AGE was performed as described before [14, 43]. In brief, mitochondria were resuspended in 1x sample buffer (0.5× TBE, 10% glycerol, 2% SDS, and 0.0025% bromophenol blue) and loaded onto a vertical 1.5% agarose gel (Bio-Rad). After electrophoresis in the running buffer (1× TBE and 0.1% SDS) for 45 min with a constant voltage of 100 V at 4°C, the proteins were transferred to Immobilon membrane (Millipore) for immunoblot.

## Supporting information

S1 Fig Identification of GPATCH3 as a negative regulator of SeV-triggered induction of type I IFNs. (A) 293T cells (1 x 10^5^) were individually transfected with ~10000 human cDNA clones (0.05 μg) together with the IFN-β reporter (0.05 μg). Twenty hours after transfection, cells were left uninfected or infected with SeV for 12 hours before luciferase assays were performed.
(TIF)

S2 Fig Knockdown of GPATCH3 enhances the activation of RLR-mediated signaling. (A) 293T cells (1 x 10^5^) were transfected with the control- or GPATCH3-shRNA plasmids (0.25 μg) and the indicated reporters (0.05 μg each). Twenty-four hours later, cells were reconstituted with an empty vector or the shRNA-resistant Flag-GPATCH3 (mGPATCH3) (0.1 μg). Twenty-four hours after reconstitution, cells were left uninfected or infected with SeV for 12 hours before luciferase assays were performed. (B) 293T cells (1 x 10^5^) were cotransfected with the indicated reporters (0.05 μg each), expression plasmids (0.05 μg each) and empty vectors or GPATCH3 expression plasmids (0.05 μg). Luciferase assays were performed 24 hours after transfection. (C&D) 293T cells (1 x 10^5^) were transfected with the control- or GPATCH3-shRNA plasmids (0.25 μg each). Thirty-six hours later, cells were left untreated, transfected with low molecular weight poly(I:C) (1 μg/ml), or transfected with high molecular weight poly(I:C) (1 μg/ml). Twelve hours later, luciferase assays were performed for the indicated reporters. (E) 293-TLR3 cells (1 x 10^5^) were transfected with the control- or GPATCH3-shRNA plasmids (0.25 μg each) together with the indicated reporters (0.05 μg each). Thirty-six hours later, cells were left untreated or treated with poly(I:C) (30 μg/ml) for 12 hours before luciferase assays were performed. Graphs show mean ± SD. n = 3. *P<0.05, **P<0.01 (Student’s *t*-test).
(TIF)

S3 Fig Overexpression of GPATCH3 enhances the replication of RNA virus. (A) 293T cells (4 x 10^5^) were transfected with empty vectors or GPATCH3 expression plasmids (1 μg). Twenty-four hours later, cells were left uninfected, infected with SeV for 24 hours or infected with VSV for 36 hours. Cell lysates were analyzed by immunoblotting with antibodies against indicated virus proteins. β-actin was used as a control.
(TIF)

S4 Fig Subcellular localization of GPATCH3. (A) Wild-type or GPACH3-deficient cells (2 x 10^6^) were left uninfected or infected with SeV for the indicated times. In the left panel, the cells were fractionated and the cytosolic and nuclear fractions were equilibrated to equal volumes and analyzed by immunoblotting with the indicated antibodies. In the right panel, the cells were fractionated and the mitochondrial and cytosolic fractions were equilibrated to equal volumes and analyzed by immunoblotting with the indicated antibodies. Cyto, cytosol; Nuc, nucleus; Mito, mitochondria.
(TIF)

S5 Fig The effects of GPATCH3 on VISA aggregation. (A) 293T cells (2 x 10^6^) were cotransfected with Flag-VISA (3 μg), HA-VISA (3 μg), together with empty vectors or GPATCH3 expression plasmids (1 μg). Coimmunoprecipitation and immunoblotting were performed with the indicated antibodies. (B) Wild-type or GPACH3-deficient cells (2 x 10^6^) were left uninfected or infected with SeV for the indicated times. The mitochondrial extracts were analyzed by SDD-AGE (top panel) and SDS-PAGE (bottom panel).
(TIF)
