# Supplementary figures and images for: GPATCH3 negatively regulates RLR-mediated innate antiviral responses by disrupting the assembly of VISA signalosome
Source: PLoS Pathog. 2017 Apr 17;13(4):e1006328. doi: 10.1371/journal.ppat.1006328 (PMC5407853; doi:10.1371/journal.ppat.1006328)

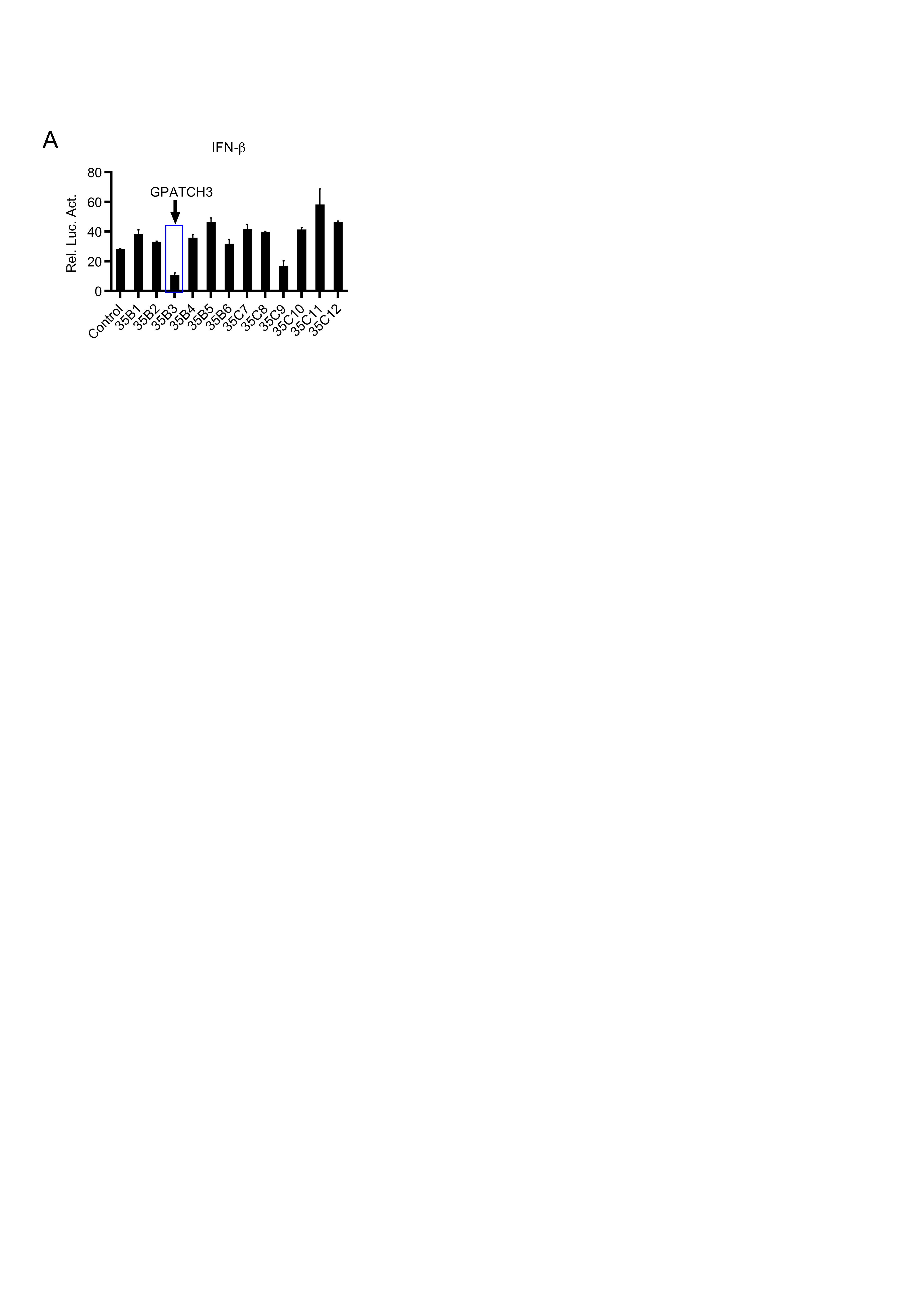

Supplement: S1 Fig — (A) 293T cells (1 x 10^5^) were individually transfected with ~10000 human cDNA clones (0.05 μg) together with the IFN-β reporter (0.05 μg). Twenty hours after transfection, cells were left uninfected or infected with SeV for 12 hours before luciferase assays were performed. (TIF) [file ppat.1006328.s001.tif]

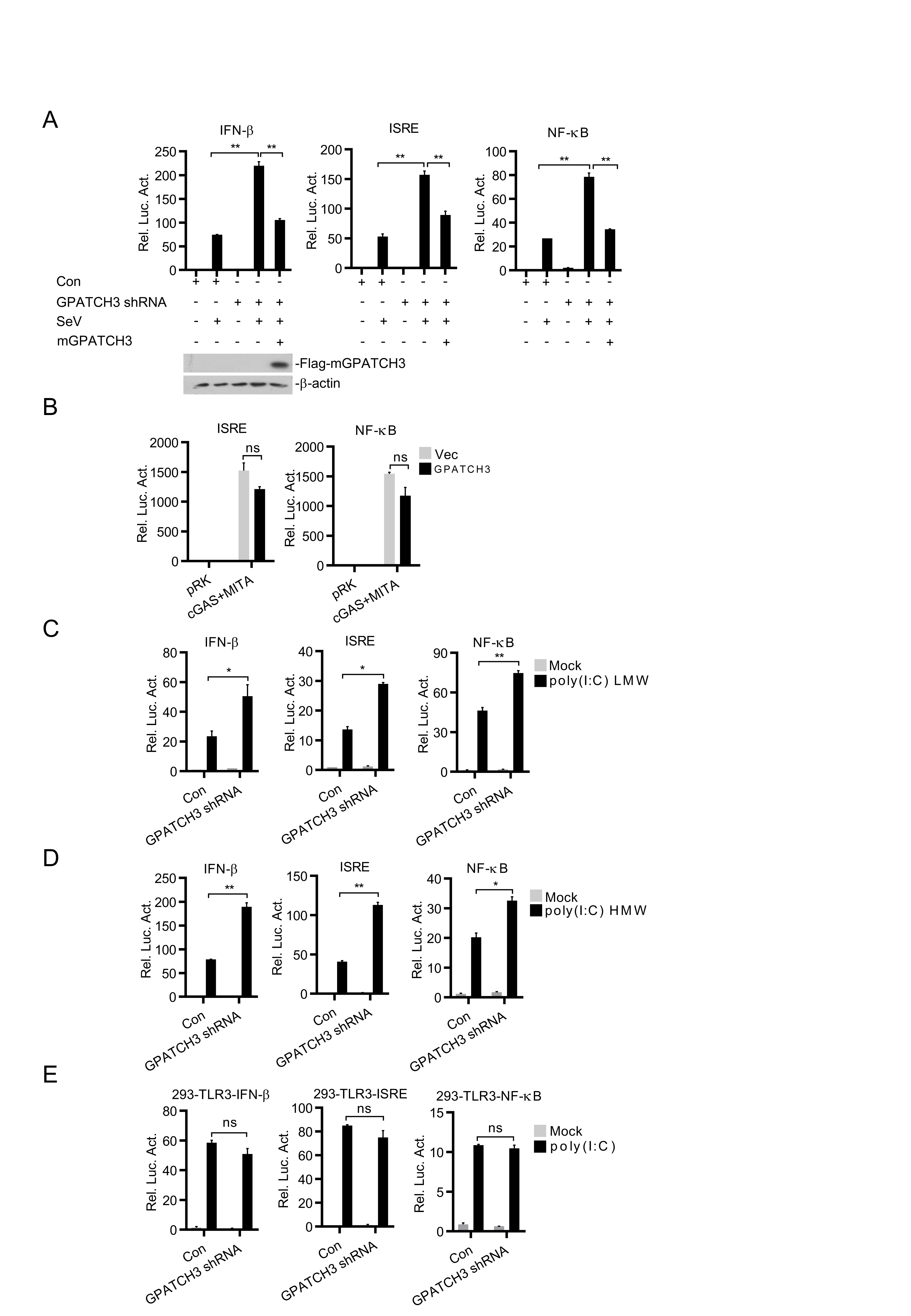

Supplement: S2 Fig — (A) 293T cells (1 x 10^5^) were transfected with the control- or GPATCH3-shRNA plasmids (0.25 μg) and the indicated reporters (0.05 μg each). Twenty-four hours later, cells were reconstituted with an empty vector or the shRNA-resistant Flag-GPATCH3 (mGPATCH3) (0.1 μg). Twenty-four hours after reconstitution, cells were left uninfected or infected with SeV for 12 hours before luciferase assays were performed. (B) 293T cells (1 x 10^5^) were cotransfected with the indicated reporters (0.05 μg each), expression plasmids (0.05 μg each) and empty vectors or GPATCH3 expression plasmids (0.05 μg). Luciferase assays were performed 24 hours after transfection. (C&D) 293T cells (1 x 10^5^) were transfected with the control- or GPATCH3-shRNA plasmids (0.25 μg each). Thirty-six hours later, cells were left untreated, transfected with low molecular weight poly(I:C) (1 μg/ml), or transfected with high molecular weight poly(I:C) (1 μg/ml). Twelve hours later, luciferase assays were performed for the indicated reporters. (E) 293-TLR3 cells (1 x 10^5^) were transfected with the control- or GPATCH3-shRNA plasmids (0.25 μg each) together with the indicated reporters (0.05 μg each). Thirty-six hours later, cells were left untreated or treated with poly(I:C) (30 μg/ml) for 12 hours before luciferase assays were performed. Graphs show mean ± SD. n = 3. *P<0.05, **P<0.01 (Student’s t-test). (TIF) [file ppat.1006328.s002.tif]

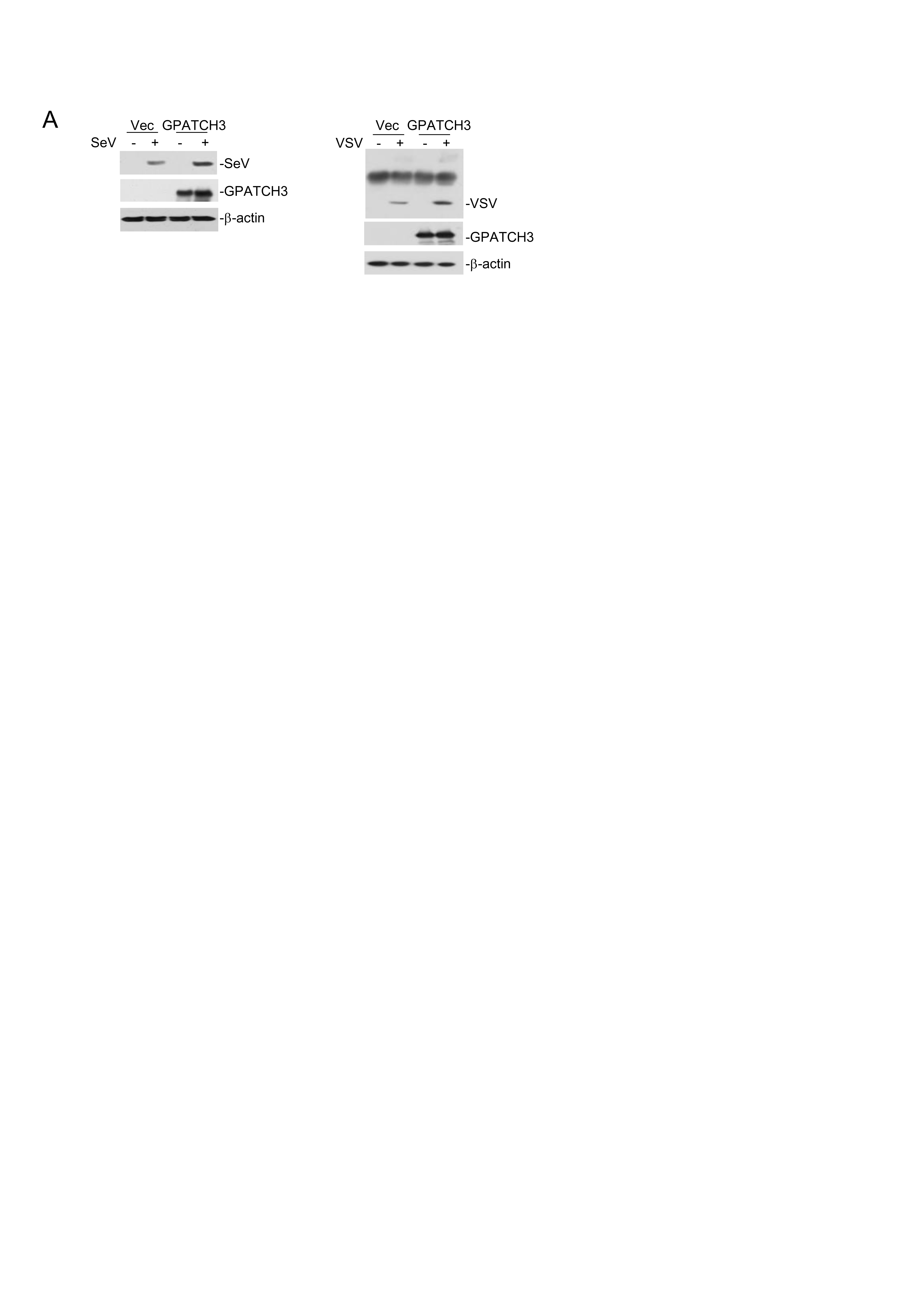

Supplement: S3 Fig — (A) 293T cells (4 x 10^5^) were transfected with empty vectors or GPATCH3 expression plasmids (1 μg). Twenty-four hours later, cells were left uninfected, infected with SeV for 24 hours or infected with VSV for 36 hours. Cell lysates were analyzed by immunoblotting with antibodies against indicated virus proteins. β-actin was used as a control. (TIF) [file ppat.1006328.s003.tif]

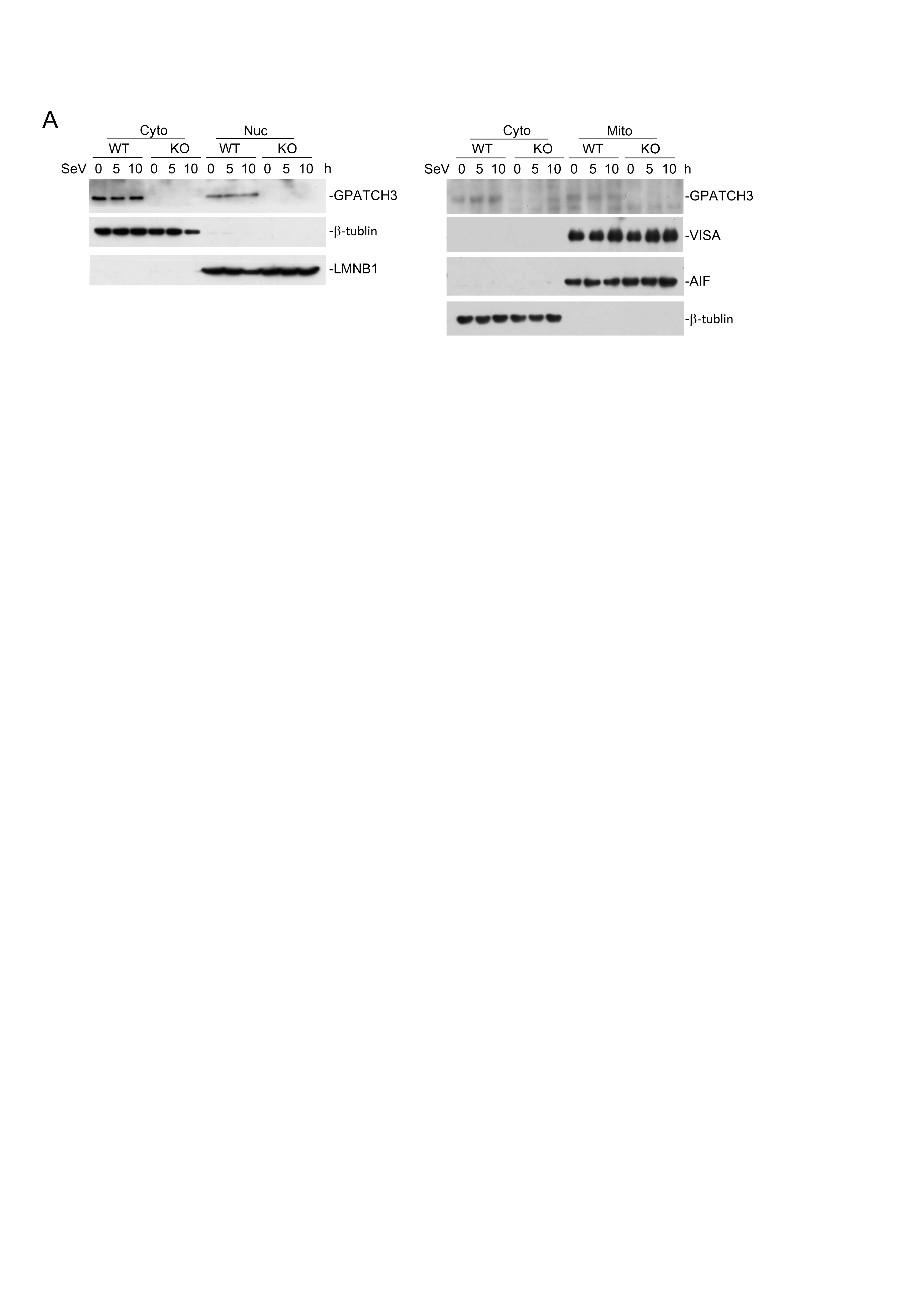

Supplement: S4 Fig — (A) Wild-type or GPACH3-deficient cells (2 x 10^6^) were left uninfected or infected with SeV for the indicated times. In the left panel, the cells were fractionated and the cytosolic and nuclear fractions were equilibrated to equal volumes and analyzed by immunoblotting with the indicated antibodies. In the right panel, the cells were fractionated and the mitochondrial and cytosolic fractions were equilibrated to equal volumes and analyzed by immunoblotting with the indicated antibodies. Cyto, cytosol; Nuc, nucleus; Mito, mitochondria. (TIF) [file ppat.1006328.s004.tif]

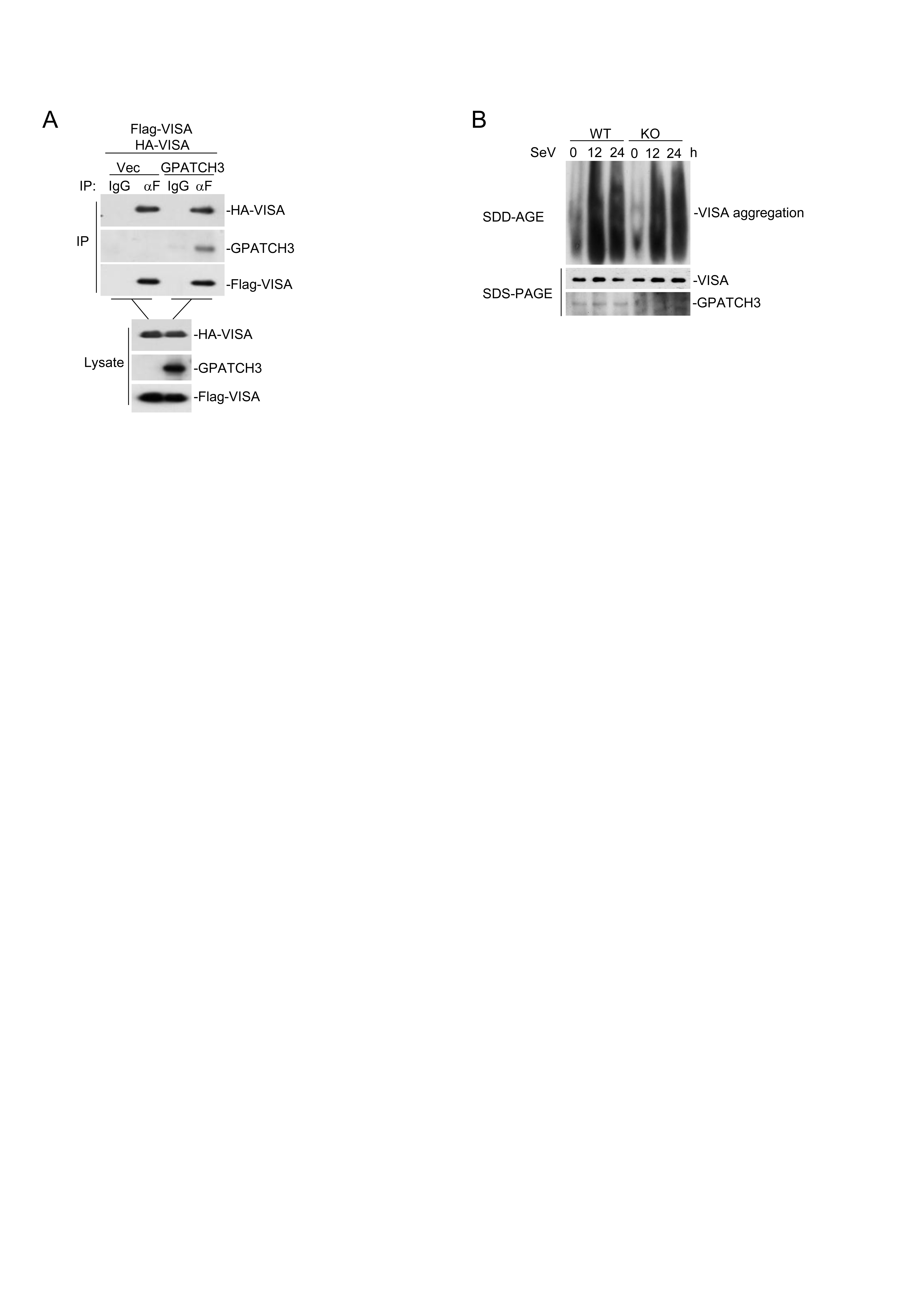

Supplement: S5 Fig — (A) 293T cells (2 x 10^6^) were cotransfected with Flag-VISA (3 μg), HA-VISA (3 μg), together with empty vectors or GPATCH3 expression plasmids (1 μg). Coimmunoprecipitation and immunoblotting were performed with the indicated antibodies. (B) Wild-type or GPACH3-deficient cells (2 x 10^6^) were left uninfected or infected with SeV for the indicated times. The mitochondrial extracts were analyzed by SDD-AGE (top panel) and SDS-PAGE (bottom panel). (TIF) [file ppat.1006328.s005.tif]
